# Genome editing for healthy crops: traits, tools and impacts

**DOI:** 10.3389/fpls.2023.1231013

**Published:** 2023-10-27

**Authors:** Kubilay Yıldırım, Dragana Miladinović, Jeremy Sweet, Meleksen Akin, Vladislava Galović, Musa Kavas, Milica Zlatković, Eugenia de Andrade

**Affiliations:** ^1^ Department of Molecular Biology and Genetics, Faculty of Arts and Sciences, Ondokuz Mayıs University, Samsun, Türkiye; ^2^ Institute of Field and Vegetable Crops, National Institute of Republic of Serbia, Novi Sad, Serbia; ^3^ Sweet Environmental Consultants, Cambridge, United Kingdom; ^4^ Department of Horticulture, Iğdır University, Iğdır, Türkiye; ^5^ Institute of Lowland Forestry and Environment (ILFE), University of Novi Sad, Novi Sad, Serbia; ^6^ Department of Agricultural Biotechnology, Faculty of Agriculture, Ondokuz Mayıs University, Samsun, Türkiye; ^7^ National Institute for Agricultural and Veterinary Research (INIAV), I.P., Oeiras, Portugal; ^8^ GREEN-IT Bioresources for Sustainability, ITQB NOVA, Oeiras, Portugal

**Keywords:** CRISPR, crops, crop improvement, pathogens, resilience, durable resistance, fungal, bacterial and virus infections, parasitic weeds

## Abstract

Crop cultivars in commercial use have often been selected because they show high levels of resistance to pathogens. However, widespread cultivation of these crops for many years in the environments favorable to a pathogen requires durable forms of resistance to maintain “healthy crops”. Breeding of new varieties tolerant/resistant to biotic stresses by incorporating genetic components related to durable resistance, developing new breeding methods and new active molecules, and improving the Integrated Pest Management strategies have been of great value, but their effectiveness is being challenged by the newly emerging diseases and the rapid change of pathogens due to climatic changes. Genome editing has provided new tools and methods to characterize defense-related genes in crops and improve crop resilience to disease pathogens providing improved food security and future sustainable agricultural systems. In this review, we discuss the principal traits, tools and impacts of utilizing genome editing techniques for achieving of durable resilience and a “healthy plants” concept.

## 1 Introduction

Crops are grown in different geographic, climatic, and agricultural conditions, where they are challenged by a vast range of pests and diseases that can substantially reduce crop yields and production (Savary et al., 2019). Managing these biotic stresses usually involves considerable effort and expense for farmers, particularly when these stressors have the ability to adapt to certain control measures. Chemical pesticides provide a level of protection, but often reliance on them is unsustainable due to resistance development, and environmental concerns (Lykogianni et al., 2021). Reduction of the efficacy of pesticides due to rapid pathogen evolution and resistance development by adaptation under selection pressure has been extensively documented for chemical pesticides (McDonald, 2014; Akın et al., 2023). Although the use of antagonistic microorganisms for biological control has advanced significantly, there are still few approved biofungicides in the market due to issues with their effectiveness, legislation, and registration procedures (Collinge et al., 2022). An efficient and alternative method to protect crops from pests and diseases is the cultivation of resistant plant genotypes in agriculture (Gvozdenac et al., 2022; Kavas et al., 2023).

Viruses, bacteria, filamentous pathogens (fungi and oomycetes) and parasitic weeds are the major groups of plant pathogens that can affect crops both in the field and post-harvest (Strange and Scott, 2005). The effects of these biotic threats on agricultural production range from none or mild symptoms to pandemics that seriously compromise crop production over large cultivation areas. Plant pathogens can be introduced into new areas through various means, such as contaminated plant material, infected seeds, soil, or infected tools and equipment. International trade and transportation of agricultural products can also facilitate the movement of pathogens across regions. (Bisht et al., 2019). Understanding the specific characteristics and modes of transmission of a particular plant pathogen is essential for developing effective bredding strategies to prevent its spread and manage diseases in agricultural and natural settings. Integrated pest management (IPM) approaches that combine with plant defense mechanisms are often used to mitigate the impact of plant pathogens and minimize their spread (Kocmánková et al., 2009).

‘Healthy plants’ are vital to sustainable and profitable crop production and to the quality and cost of the nation’s supply of food, fuel, and fiber. However, maintaining “healthy plants” is a challenge due to climate and other environmental changes that can disrupt the interactions between species (Tamura et al., 2022) in a range of environments (Karavolias et al., 2021). Currently, climate change is favoring enlargement of the geographical distribution of some already existing and newly emerging pests and invasive plants (Jones and Barbetti, 2012; King et al., 2018). Furthermore, the markets and economy require extensive movement of plants and agricultural goods between continents, facilitating the movement of pathogens, along with human activities such as travel and urbanization that promote the entry of new pathogens into agricultural ecosystems compromising crop health (Franic et al., 2022).

Crop cultivars in commercial use have often been selected because they show high levels of resistance to pathogens. However, widespread cultivation of these crops for many years in the environments favorable to a pathogen requires durable forms of resistance to maintain “healthy plants”. This durable resistance depends on the variability of pathogenicity, and the nature of the resistance mechanisms in crop cultivars (Nnadi and Carter, 2021). Many pathogens are heterozygous many different pathotypes or races which can rapidly adapt to new environments or hosts. Some pathogens are host-specific, whereas others have a diversity of hosts and thus can maintain reservoirs of infective pathotypes with a greater ability to evolve and adapt to climate and weather conditions (Amari et al., 2021).

Breeding of new varieties tolerant/resistant to biotic stresses by incorporating genetic components related to durable resistance, developing new breeding methods and new active molecules, and improving the IPM strategies have been of great value, but their effectiveness is challenged by the newly emerging diseases and the rapid change of pathogens due to climatic changes (Hussain, 2015; Bhoi et al., 2022). Achieving continuous production of resistant varieties needs continuous adjustment of breeding methods. Hence, in recent years, novel methods to enhance genetic resistance have been developed. These include changing the genetics of crop plants, introducing novel genes into plants and the expression of interfering RNAs (RNAi). Developing disease-resistant crops through genome editing-based techniques offers an effective, environmentally friendly, low-input, and sustainable approach to plant disease management (Ali et al., 2022). Their effective application has been supported by the characterization of many immune receptors, (“*R*” genes) and the genetic basis of cell surface immunity in the last decades. This progress has enabled us to understand the molecular basis of interactions between plants and pathogens and plant innate immunity, both of which are essential for developing disease-resistant plant varieties (Ali et al., 2022; Bhoi et al., 2022).

Genome editing (GE) technology has developed new tools and methods to identify genes involved in defence in crops and to increase crop resilience to pathogens thus providing improved food security within agricultural systems that are more sustainable (Gosavi et al., 2020). Here we review the main traits, tools and impacts of the application of genome editing techniques in crop improvement for the achievement of durable resilience and a “healthy crops” concept.

## 2 Cross-talk between plants/pathogens via plant immunity

**Figure 1:**
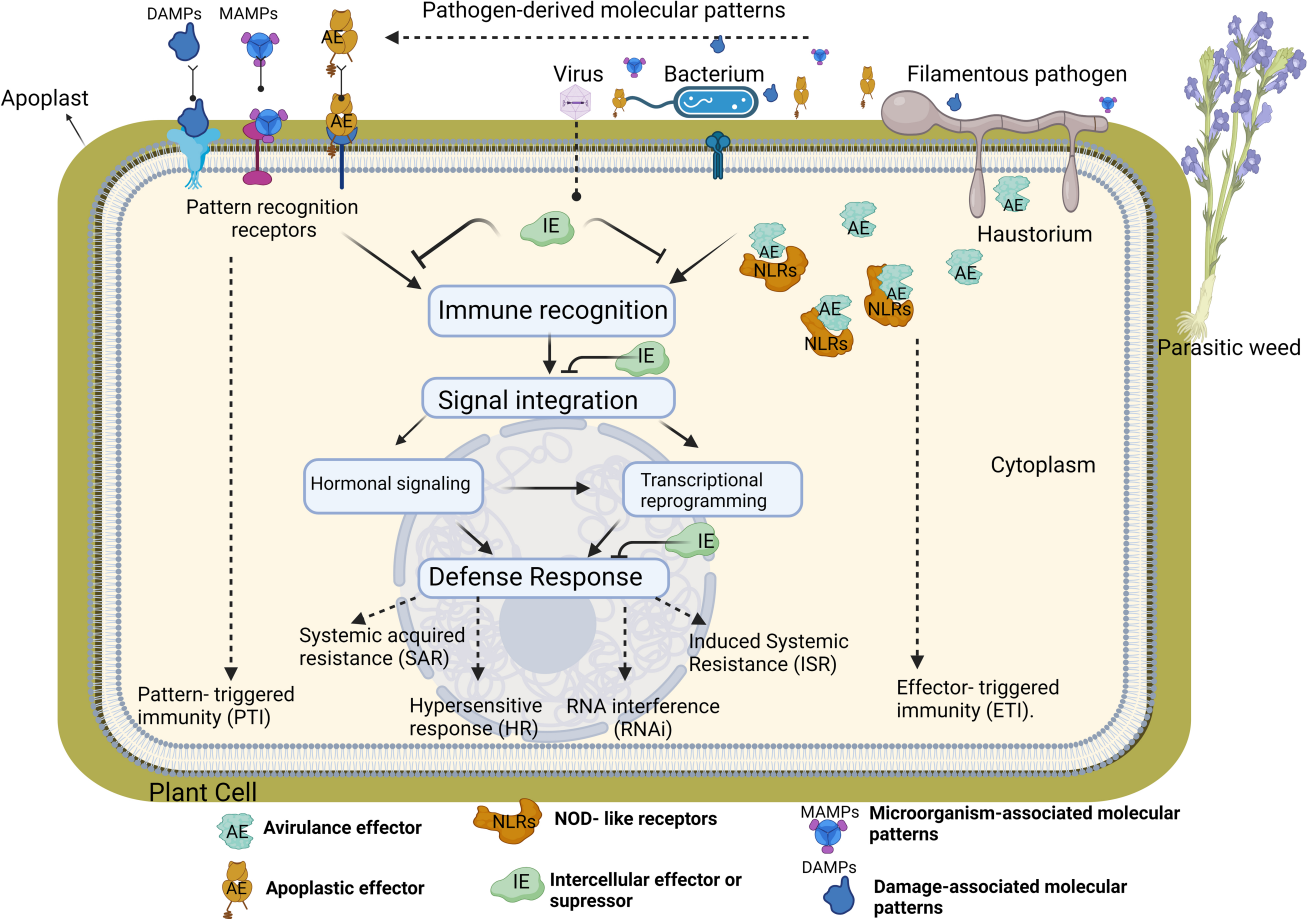
The activation of the plant innate immune system requires tree steps; immune recognition, signal integration and defense response. Plants use numerous cell surface and intracellular immune receptors to recognize microorganism/host-derived molecular patterns (MAMPs and DAMPs), or apoplastic/avirulance effectors (AE). Cell surface pattern recognition receptors (PRRs) bind to MAMPs or DAMPs or AE directly through their extracellular domain while NOD- like receptors (NLRs) recognize effectors delivered inside host cells by directly binding effectors or sensing modulation of effector host targets. PRR- mediated recognition of MAMPs or DAMPs elicits pattern- triggered immunity (PTI), and NLR- mediated pathways trigger effector-triggered immunity (ETI). Activation of immune receptors subsequently initiates the second phase of immune system. In this phase, various immune signaling events such as calcium fluxes, activation of mitogen- activated protein kinase (MAPK) cascades, alteration of host transcription and phytohormone signaling trigger the defense response in each cellular compartment in plants. Hormone- dependent response generally activates a large set of plant defense-related genes against biotrophs. For instance, hormone accumulation in plants triggers hypersensitive response (HR) which cause rapid local death of the infected and surrounding cells to restrict the spread of pathogens to other parts of the plant. Accumulation of hormones and pathogenesis-related proteins in the plants can also induce long-lasting protection against a broad spectrum of pathogens, called systemic acquired resistance (SAR). In this resistance, putative SAR signal molecules move from the infected systemic organs to non-infected distant parts of the plant where it make more resistance to pathogens prior to infection. Induced Systemic Resistance (ISR) is another defense response increasing physical or chemical barriers of the host plant against pathogens rather than direct killing or inhibiting the invading pathogen. RNA interference (RNAi) is the last plant resistance mechanism activated during the viral infection.

Plants and pathogens have an endless complex co-evolutionary arms race where pathogens try to overcome plant defenses and in turn, plants have developed a range of defence mechanisms to detect and prevent pathogen invasion (“zig-zag model”) (Jones and Dangl, 2006). Throughout evolution, plants have been armed with several physical barriers and biochemical adaptations to prevent the entry of pathogens into the plant cells. In addition, the plant immune system has been developed according to the complexity of the feeding behaviors of pathogens through co-evolution over millions of years (Voigt, 2014). Plant pathogens can be biotrophs that completely or partially rely on host cells for the completion of their life cycle. These types of pathogens manipulate the host metabolism to induce favorable nutritional conditions and maintain host viability to acquire nutrients as much as possible. Biographies cause relatively minor damage to the host plant cell, while necrotrophic pathogens kill their hosts during infection by using all their sources. Plants do not have an adaptive immune system due to their lack of specialized immune cells. Nevertheless, plants developed resistance to biotrophs and necrotrophs with induced signal transduction routes that share cross-talk and independent pathways. This plant’s innate immune system is based on pathogen receptors detecting the presence of pathogens (immune recognition) and molecular signalling pathways to transmit the message of invasion (signal integration) to the cell nucleus (Andolfo and Ercolano, 2015). Signal integration of invasion alters the transcriptional gene expression in the nucleus and activates the defence response in host plant cell (**Figure 1**).

### 2.1 Plant innate immunity

If a pathogen manages to enter a host, a multi-layered innate immune system is activated as a defense response (Jones and Dangl, 2006; Andolfo and Ercolano, 2015). The first layer of the defense comprises receptor-like proteins or receptor-like protein kinases known as plant/pathogen recognition receptors (PRRs) that detect pathogens at the plant cell membrane surface and in the apoplast (Wise et al., 2007; Dodds and Rathjen, 2010). At the plant cell membrane, PRRs “recognize” conserved microbial elicitors known as microbe-associated molecular patterns (MAMPs, also referred to as pathogen-associated molecular patterns-PAMPs), and pathogen proteins (apoplastic effectors) that are produced in the apoplast and this initiates a plant defence response called MAMP/PAMP-triggered immunity (MTI/PTI) (Jones and Dangl, 2006; Thomma et al., 2011; Andolfo and Ercolano, 2015; Boschi et al., 2017; Boutrot and Zipfel, 2017). Moreover, the pathogen attack can trigger plant signals called Damage-Associated Molecular Patterns (DAMPs) that can also activate PTI (Hou et al., 2019). The MAMPs/PAMPs include several components: bacterial flagellin, elongation factor thermo-unstable (EF-Tu), and fungal chitin, whereas DAMPs are molecules that are released from damaged cells undergoing pathogen invasion (**Figure 1**) (Lanna-Filho, 2023). As the battle continues, the plant produces reactive oxygen species (ROS) and secrets antimicrobial products such as phytoalexins, and phenolic compounds like flavonoids and tannins in the intercellular spaces that can destroy pathogens (Doehlemann et al., 2008; Saijo et al., 2018; Kebert et al., 2022)

In the cytoplasm, the pathogen secret proteins (cytoplasmic effectors, formerly known as avirulence factors) that target plant susceptibility (*S*) genes to manipulate plant processes to support pathogen growth, promote disease development and induce susceptibility. This phenomenon is called effector-triggered susceptibility (ETS) (Jones and Dangl, 2006; Jones and Barbetti, 2012; Weßling et al., 2014; Hui et al., 2019). As a counter defence strategy, proteins encoded by disease resistance genes (*R* genes) recognize pathogen effectors (Zhang et al., 2017; Collinge, 2020). Most R proteins contain domain-rich amino acid leucine (leucine-rich repeat - LRR), and have a nucleotide binding site (NBS) and NOD-like receptors (NLRs) (**Figure 1**). The recognition of the pathogen effectors by plant *R* genes initiates NLR-mediated response known as NLR or effector-triggered immunity (NTI/ETI) to stop pathogen growth and development (Jones and Dangl, 2006; Dodds and Rathjen, 2010; Win et al., 2012; Lo Presti et al., 2015., Jones et al., 2016). This plant immunity response is generally stronger than pathogen-triggered immunity (PTI) (Jones and Dangl, 2006). ETI results in events like cell wall modifications (e.g. depositions of lignin and callose), stomata closure, expression of pathogenesis-related genes that induce production of proteins that show antimicrobial activity (e.g., chitinases, β 1-3 glucanases, defensins, peroxidases), secondary metabolites like phytoalexins and the accumulation of plant hormones related to plant defence, including salicylic acid (SA), jasmonic acid (JA) and ethylene (ET) (Mukhtar, 2013; Uehling et al., 2017; Andersen et al., 2018). The most extreme consequences of ETI include a hypersensitive response (HR) along with the generation of ROS that leads to programmed cell death (PCD) and the formation of necrotic lesions, where the infected plant cells kill themselves to protect other cells and restrict the spread of the pathogen from the infection site to neighboring cells (**Figure 1**) (Gong et al., 2019). Moreover, recently, Khattab et al. (2023) identified trans-ferulic acid, a monolignol precursor as a “plant surrender signal” that accumulates in grapevines under stress. The ferulic acid activates the secretion of the fungal phytotoxin fusicoccin A aglycone which stimulates programmed cell death after infection with the pathogenic necrotrophic fungus *Neofusicoccum parvum*.

### 2.2 RNAi and R gene-mediated plant immunity

Two key components have been described for plant-virus interactions and plant defence responses to viral pathogens; RNA silencing and R gene-mediated pathways. RNA gene silencing [also called RNA interference (RNAi)] is the main plant defence response to viral pathogens (Moon and Park, 2016). Most plant viruses have RNA genomes that contain a regulatory stem-loop. These loops are recognized by virus-encoded RNA-dependent RNA polymerases to copy the viral genome into complementary double-stranded RNAs (dsRNAs) (Ruiz-Ferrer and Voinnet, 2009). Host ribonuclease III-like protein, also called Dicer-like (DCL), recognizes the dsRNAs and then breaks them up into short interfering RNAs (siRNAs). The siRNAs (20-25 bp in length) have complementary sequences to the viruses and act as guides to direct RNA-induced silencing complex (RISC) in their target and degrade the viral RNA molecules (Mallory et al., 2008; Ruiz-Ferrer and Voinnet, 2009). Interestingly, plant viruses often encode viral suppressor RNAi (VSRs) to inactivate the plant RNAi-mediated silencing pathway and enhance viral replication, assembly, or movement (Ding and Voinnet, 2007). VSR-mediated suppression of antiviral RNA silencing pathway is known to occur in two ways. VSRs can sequester the small RNA duplexes to block their binding to viral dsRNAs (Lakatos et al., 2006) or directly impede the activity of RISC proteins to impair the assembly of the complex (Carbonell and Carrington, 2015). Besides RNAi, plants have also developed a second layer of dominant and recessive defence against viruses via resistance genes (*R*-genes) (De Ronde et al., 2014). Most of these *R*-genes are triggered by a virus and confer dominant resistance like in Ty-1 *R*-gene from tomato against tomato yellow leaf curl virus (TYLCV). This gene encodes an RNA-dependent RNA polymerase and confers resistance against TYLCV by amplifying the RNAi signal (Verlaan et al., 2013). Since viruses require host factors for their infection, cross-talk between such plant susceptibility factors and the virus may also lead to resistance (Moon and Park, 2016). For instance, some viruses encode a cap-like structure to interact with the host translation initiation factors (eIF4E/eIF4G) for the expression of the viral genome. Loss of function in these factors leads to a recessive resistance in plants (Truniger and Aranda, 2009). Indeed, viral pathogens generally encode proteins for the suppression of plant’ RNAi defence mechanisms (Wang et al., 2012). Therefore, both RNAi and *R* gene-mediated pathways in plants undergo crosstalk to maximize the efficiency of defence responses against viral infections (Nakahara and Masuta, 2014). For example, in the *Arabidopsis* hypersensitive response to the turnip crinkle virus, the *HRT* genes respond to the TCV coat protein by producing a DNA-binding protein. HRT-mediated resistance requires double-stranded RNA-binding protein-4 which is also the component of the RNAi (Zhu et al., 2013) PTI also limits virus infection in plants and this defence response is mediated by dsRNA (Niehl and Heinlein, 2019).

### 2.3 Hormone-mediated immunity and crosstalk between plants and pathogens

Activation of PTI and ETI in infected tissues often triggers a third layer of plant immunity referred to as induced resistance (IR) and can occur at the site of the attack, in parts of plants distal from the site of infection, or throughout the entire plant (**Figure 1**). During those systemic immune responses, hormonal interactions and their signalling pathways play the role of central regulators in plant defence against a wide range of pathogens and insects (Berens et al., 2017). Different hormones accumulate in plant tissues depending on the type of attacker and each hormone regulates its own immune network. Salicylic acid (SA) and Jasmonic acid (JA) are the two basic hormones forming the backbone of plant immune systems against pathogens and insects (Wasternack and Song, 2017; Zhang and Li, 2019). The SA-dependent response generally activates a large set of plant defence-related genes against biotrophs (Vos et al., 2015). For instance, SA accumulation in plants triggers a rapid local death of the infected and surrounding cells to restrict the spread of pathogens to other parts of the plant (Balint-Kurti, 2019). In addition to this rapid hypersensitive response, the accumulation of SA and pathogenesis-related proteins in plants can also induce long-lasting protection against a broad spectrum of microorganisms and insects, called systemic acquired resistance (SAR) (Backer et al., 2019). In this resistance, putative SAR signal molecules such as methyl salicylate move from the infected systemic organs to non-infected distant parts of the plant where it induces pathogenesis-related genes against pathogens. In this way, distant leaves or tissues become more resistant to pathogens before infection (Backer et al., 2019; Balint-Kurti, 2019). Induced Systemic Resistance (ISR) is another resistance strategy in plants that is activated by infection. This strategy depends on increasing the physical or chemical barriers of the host plant against pathogens rather than directly killing or inhibiting the invading pathogen. Plants are sensitized to produce an enhanced ISR response by infection with beneficial bacteria and fungi living in the rhizosphere and signal transduction pathways activated by JA (Yu et al., 2022). These root-associated mutualistic microbes boost plant defenses, rendering the entire plant more resistant to pathogens and pests (Backer et al., 2019; Yu et al., 2022).

JA and its oxylipin derivatives (jasmonates) are generally synthesized and accumulated in plants in response to herbivore arthropods or infection with necrotrophs (Wang et al., 2021). Some herbivore insects take their nutrients from plants by mechanical damage of plant tissues while necrotrophs derive their energy from dead or dying cells (Vega-Muñoz et al., 2020). During the insect chewing or wounding during herbivory, JA is rapidly synthesized locally on the damaged part of the plant and systemically in parts of plants not affected by pathogens (Wang et al., 2021). This increase in JA concentration activates the expression of defense-related genes that induce production of toxic secondary metabolites, formation of physical barrier (such as trichome) and generation of volatile organic compounds (VOCs) (Escobar-Bravo et al., 2017; Vega-Muñoz et al., 2020). However, some biotrophic pathogens and hemibiotrophic pathogens develop mechanisms to evade this JA-mediated plant defense through injecting toxins and virulence-effector proteins into host cells to suppress JA signaling components (Vargas et al., 2012; Wang et al., 2021). SA and JA can act alone or show synergistic and antagonistic interactions with each other or with other hormones in a complex interplay (Liu et al., 2016). This phenomenon is known as hormone crosstalk and is an important component of the architecture of the plant immune signaling network (Yang et al., 2019). For instance, JA pathway is divided into two branches (Pieterse et al., 2014; Yıldırım and Kaya, 2017). The ERF branch of the JA pathway is co-regulated by ethylene (ET). This branch is activated by infection with necrotrophic pathogens. The second branch of JA pathway, MYC branch, is co-regulated by abscisic acid (ABA) to provide protections against chewing insects (Aerts et al., 2021). It has also been shown that JA signaling can block SA accumulation in plants through modulation of multiple transcription factors (Caarls et al., 2015). This crosstalk between JA and SA signaling pathways has been reported to coordinately regulate plant disease resistance against necrotrophic or hemibiotrophic pathogens (Yang et al., 2015). SA is generally known to activate the expression of early defense-related genes, while JA induces late defense-related gene expression in infected plants (Caarls et al., 2015; Yang et al., 2015; Sucu et al., 2018; Aerts et al., 2021).

## 3 Tools for crop genome editing and introduction of durable resilience

Because plants and their pathogens have been evolving together, they have developed a sophisticated mode of communication where changes in virulence of the pathogen is being balanced by the changes in the resistance of the host, and vice versa. This plant-pathogen balance is known as “gene-for-gene concept” and it is an integral part of the plant’s and pathogen’s life cycle. The concept in which a single gene of the host corresponds to the single gene of the pathogen has proven extremely important in plant breeding (Hammond-Kosack and Jones, 1997; Zaidi et al., 2018; Naidoo et al., 2019; Li et al., 2020; Rato et al., 2021). However, it is a quite complex interaction since both plants and pathogens can have multiple genes that can affect their resistance and virulence, respectively and there are many different races of a single pathogen species that can infect different plant cultivars depending on the combination of their resistance genes. Pathogen strains that can induce resistance reaction in a plant have evolved dominant avirulence (Avr) genes, and as counter defence strategy plants have evolved dominant resistance (R) genes. In contrast, pathogen strains that can “sneak” by the plant’s defensive system undetected have recessive virulence genes and these strains can cause the disease. The weakness of R-mediated resistance leads to the emergence of resistant pathogen strains and thus it is short-lived in the field (Zaidi et al., 2018; Li et al., 2020; Tyagi et al., 2020; Pan et al., 2021; Rato et al., 2021). Moreover, this type of resistance is associated mainly with biotrophic and hemibiotrophic pathogens, whereas it is challenging to use these resistant strategies against necrotrophic pathogens due to their need to colonies the dead tissue (Collinge and Sarrocco, 2022).

**Figure 2:**
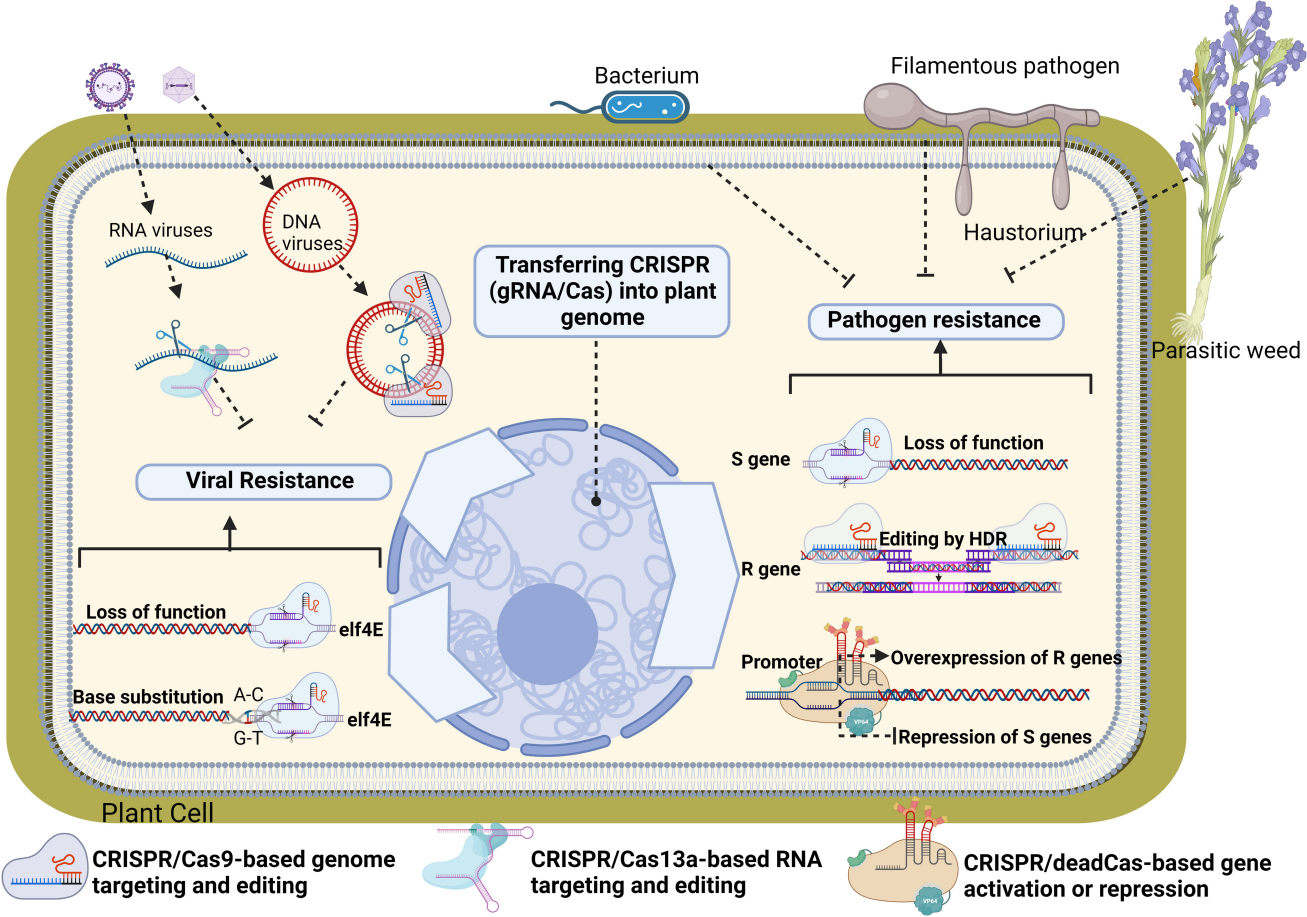
Theoretical and already tested CRISPR/Cas applications to increase plant resistance toward pathogens. CRISPR/Cas9 can be used to disrupt plant susceptibility (S) genes (such as Eukaryotic translation initiation factor 4E (elf4E)) by targeting coding regions to knock out these genes, or to alter sequences of promoter regions, precluding pathogen effector binding to the promoter and thus disrupting plant susceptibility. In addition, Dead Cas9-based CRISPR systems could be used to overexpression of resistance genes or suppression of S genes. CRISPR-mediated homology-directed repair (HDR) can be used to introduce resistance (R) genes against pathogens in cases where the plant-pathogen interaction (and S genes) is not well studied. To develop pathogen resistance without disrupting or replacing whole genes, CRISPR based base-edition technology can be used to achieve specific mutations (biomimicking) in genes to turn them into resistant genes against pathogens of interest. The native function of CRISPR can be also mimicked directly to target and interfere with the genomes of pathogens of interest without affecting plant genome. For example, CRISPR can interfere with DNA genomes of viruses through DNA-targeting gRNA/Cas9 systems or it can disrupt pathogen’s RNA genomes through RNA-targeting gRNA/Cas13a systems. Loss of function in S genes.

Currently, several crop plants have fully sequenced genomes and these annotated genomes result in increased knowledge of the molecular details and genetic functions of plant genes. This knowledge is exploited by genome editing (GE) innovations creating a greater advancement in understanding the gene regulatory functions in plants, pathogens and their interactions. GE has been accepted as a new breeding technique and has been used to improve plant resistance against many kinds of pathogens in past decade (Secgin et al., 2022). GE techniques such as zinc-finger nucleases (ZFN), transcription-activator-like effector nucleases (TALEN) use DNA nucleases guided with the engineered proteins. On the other hand, newly discovered CRISPR/Cas system depend on oligo-directed mutagenesis with sequence-specific nucleases. Due to its high accuracy, cost-effectiveness, and simplicity, the CRISPR/Cas9 system became the most popular GE tool for plant breeding (Aksoy et al., 2022). This system consists of the Cas protein inducing a double-strand break (DSB) in the DNA, and the single guide RNA (sgRNA) directing the Cas protein to the genomic target. The specificity of the system is conferred by easily programmable 20-nt-long guide RNA sequences complementary to the target genomic sequence (Cardi et al., 2023). CRISPR/Cas-mediated DSB can result in insertions or deletions (InDels) in the target DNA when repaired by the error-prone non-homologous end joining (NHEJ) mechanism. This would result in a simple random mutation in the target gene, most likely leading to a frameshift causing a loss-of-function phenotype. (**Figure 2**). CRISPR/Cas could be also used for the introduction of a sequence of choice via homology-directed repair (HDR) with the presence of a repair template in the complementary flanking arms. Such editing of the original gene sequence by introducing specific mutations can also be used to alter a single nucleotide in the genome to change the amino acid structure of the proteins, enzyme activities or substrate specificity [(**Figure 2**) Miladinović et al., 2021]. In recent years, dead or deactivated Cas (dCas)-based technologies have been developed and used for alteration of gene expression in plants. One of this technology is known as CRISPR activation or CRISPRa in which a catalytically dead (d) Cas9 is fused with a transcriptional effector to modulate target gene expression. Once the guide RNA navigates to the genome locus along with the effector arm, the dCas9 is unable to cut, and instead, the effector activates the downstream gene expression. On the contrary, CRISPR interference or CRISPRi technology just contains a catalytically dead (d) Cas9 and when guide RNA navigates to the genome locus along with the effector arm, it represses the downstream gene expression instead of activating it. In this section already tested genome editing approaches used to increase plant resistance toward pathogens will be listed and summarized with some theoretical applications.

### 3.1 Genome editing for viral resistance

Plant DNA and RNA virus families cause diseases and crop losses in a broad range of important crops. The dsDNA nature of DNA viruses, *Geminiviridae* and *Caulimoviridae*, make them good targets for CRISPR/Cas and this has become a popular approach to antiviral engineering in crops (**Table 1**). CRISPR-mediated resistance against DNA viruses was developed for *Cauliflower mosaic virus* (Liu et al., 2018), *Cotton leaf curl Multan virus* (Yin et al., 2019), Tomato yellow leaf curl virus (TYLCV), (Ali et al., 2015a; Ali et al., 2015b; Seçgin et al., 2021) *Beet severe curly top virus* (Baltes et al., 2015) and *Bean yellow dwarf virus* (Ji et al., 2015) in *Arabidopsis* and tobacco plants. gRNA/Cas9 constructs were designed to target and cleave viral replication (REP), coat protein and noncoding stem-loop sequences [TAATATTAC] common to all geminiviruses. Transient and stable expression of these constructs in transgenic plants exhibited high levels of viral resistance with significant reductions in virus accumulation and disease symptoms and revealed that the strongest virus inhibitory effect was achieved by the gRNA targeting the stem-loop sequence (Ali et al., 2015a; Baltes et al., 2015; Ji et al., 2015; Yin et al., 2019). This indicated that the stem-loop region could be a good target in CRISPR/Cas9-mediated resistance for broad-spectrum resistance to other geminiviruses. Other findings by Ali et al. (2015b) corresponded well with this suggestion that transient expression of gRNA/Cas9 construct confers resistance against mixed infection with Beet curly top virus and *Merremia mosaic virus* (MeMV), both of which share this conserved stem-loop sequence. In another approach, catalytically inactivated Cas9 (deadCas9) was successfully targeted to conserved stem-loop sequence of *Cotton leaf curl virus* to inhibit its replication and accumulation (Khan et al., 2019). In addition to model plants, CRISPR-mediated resistance against DNA viruses has also been carried out on sugar beet infections with *Beet Curly Top Iran Virus* (Yıldırım et al., 2022; Yıldırım et al., 2023), barley plants infected with wheat dwarf virus (Kis et al., 2019) and tomato infected with *TYLCV* (Tashkandi et al., 2018).

**Table 1:** Crop genome editing for viral resistance.

Crop	Target	Genetic Changes	Method	Status	Reference
*C. sativus.*	*Cucumber vein yellowing virus CVYV) Zucchini yellow mosaic virus (ZYMV) and Papaya ring spot mosaic virus-W (PRSMV)*	Base editing (A-G) of susceptibility factor (eIF 4E)	*Agrobacterium mediated gRNA/cas9 transfer into arabidobsis and selection of non-transgenic mutants in T3*	Broad virus resistance in non-transgenic cucumber	Chandrasekaran et al. (2016)
*H. vulgare*	*Wheat dwarf virus (WDV)*	Knockout the MP, RP, CP and IR of WDV	*Agrobacterium-mediated transient expression in barley*	Efficient viral resistance in barley	Kis et al. (2019)
*M. esculenta*	*Cassava brown streak virus (CBSV) and Ugandan cassava brown streak virus (UCBSV)*	Mutations in cassava susceptibility factor (eIF4E) of cassava	*Agrobacterium mediated stable gRNA/cas9 transfer and selection of mutants in cassava*	Suppressed disease symptoms and reduced virus titre in mutant cassava roots compared to WT	Gomez et al. (2019)
*M. esculenta*	*African Cassava Mosaic Virus (ACMV)*	Knockout in Rep and *MP* genes of ACMV	*Agrobacterium mediated stable expression of Cas9 protein together with gRNA*	Fails to confer effective resistance to ACMV in cassava and viral mutant escape	Mehta et al. (2019)
*Musa* spp.	*Banana Streak Virus (BSV)*	Targeting the ORF and IR in BSV	*Generation of transgenic banana with agrobacterium mediated gRNA/Cas9 transfer*	Full resistance in transgenic banana to endogenous BSV	Tripathi et al. (2019)
*N. benthamiana*	*Tomato yellow leaf curl virus (TYLCV)*	Knockout the viral IR, MP and REP coding region	*Stable overexpression of CAs9 in tobacco and transient expression of gRNA in tobacco*	Delayed and reduced accumulation of viral DNA, significantly attenuating symptoms of TYLCV infection in tobacco.	Ali et al. (2015b)
*N. benthamiana*	*Bean yellow dwarf virus (BeYDV)*	Knockout the viral LIR	*Transient expression assay with Agrobacterium*	Reduced virus load and disease symptoms in BeTDV treated tobacco	Baltes et al. (2015)
*N. benthamiana*	*Beet severe curly top virus (BSCTV)*	Knockout the coding and non-coding parts of BSCTV genome	*Stable and transient expression of gRNA/Cas9 in Arabidobsis and tobacco*	Strong reduction in viral load and disease symptoms in tobacco and *Arabidopsis*	Ji et al. (2015)
*N. benthamiana and* *S. lycopersicum*	*Tomato yellow leaf curl virus (TYLCV)*	Knockout the CP and Rep of TYLCV	*Agrobacterium-mediated stable gRNA/Cas9 transfer into tomato and tobacco*	Low accumulation of TYLCV in tomato and tobacco transgenic plants	Tashkandi et al. (2018)
*N. benthamiana*	*Cucumber mosaic virus (CMV) or tobacco mosaic virus (TMV)*	Targeting the RNA viruses	*Plants expressing FnCas9 and sgRNA specific for the RNA viruses*	Significantly attenuated virus infection symptoms and inheritable reduced viral accumulation in plants	Zhang et al. (2018)
*N. benthamiana*	*Chilli leaf curl virus (ChiLCV)*	Multiple targeting the genes of ChiLCV	*Agrobacterium transient assay in tobacco*	Resistant to ChiLCV with reduced viral accumulation	Roy et al. (2019)
*N. benthamiana*	*Cabbage leaf curl virus (CaLCuV)*	Knockout the viral IR and REP coding region	*Agrobacterium mediated transient expression of gRNA/Cas9 constructs*	Complete resistance to CuLCuV infection in transgenic tobacco	Yin et al. (2019)
*O. sativa*	*Rice tungro spherical virus (RTSV) and Rice tungro bacilliform virus (RTBV)*	Knockout in initiation factor 4 gamma gene (*eIF4G*)	*Agrobacterium-mediated transformation of gRNA/Cas9 into rice immature embryos*	In-frame mutations in one conferred resistance to RTSV and RTBV in rice	Macovei et al. (2018)
*S. tuberosum L.*	*Potato virus Y (PVY)*	Targeting conserved regions in expressed genes of PVY strains.	*Agro-infiltration of tobacco leaves and generation transgenic potato plants with LshCas13a/sgRNA*	Suppressed PVY accumulation and disease symptoms in transgenic potato	Zhan et al. (2019)
*B.vulgaris*	*Beet curly top Iran virus(BCTIV)*	Multiple targeting of the expressed genes of BCTIV	*Agrobacterium-mediated transient expression of gRNA/Cas9 in sugar beet leaves*	Full viral resistance in sugar beet	Yıldırım et al. (2023)

All these studies indicated the successful use of CRISPR/Cas9 to enhance virus resistance in plants. However, GE-based viral resistance in plants has some limitations. For example, targeting and mutating the virus genome could create a new variant of the virus that could be more aggressive and resistant to plant defence systems. Therefore, CRISPR systems targeting the multiple promoters or gene structures need to be designed to hinder mutant viral escape and to obtain full viral resistance. In addition, the requirement of PAM and dsDNA structure for effective digestion makes it impossible to target ssDNA structure of the viruses by CRISPR (Yıldırım et al., 2023). Fortunately, the newly discovered CRISPR/Cas systems offer precise and simple solutions to these problems (preventing viral escape, multiplexing DNA targeting, and even easy viral diagnostics). For instance, CRISPR/Cas12 has been realized to be much more versatile than CRISPR/Cas9 (Ali and Mahfouz, 2021). Cas12 requires only a short crRNA (making engineering easy), can process polycistronic crRNAs (making multiplexing possible, with no chance of virus escape, allowing multiple genomic loci to be edited at once), targets ssDNAs, and dsDNAs, and degrades ssDNAs via trans activity, Cas12 is comparatively small, and can easily be delivered via deconstructed viral vectors.

Furthermore, the nonspecific degradation of ssDNAs or ssRNAs (reporters) upon recognition of a specific target by Cas12, Cas13, and Cas14 variants provides the opportunity to develop an efficient diagnostic system for deployment in the field. Coupling of the target specificity and nuclease activity of Cas variants with target enrichment (via isothermal amplification, LAMP, RPA) and signal amplification (CONAN or SENSR) has the potential to change the entire scope of plant virus detection and control measures. Discovery of RNA-targeting Cas endonucleases (FnCas9 and Cas13) offered new possibilities for controlling RNA virus infections in plants. CRISPR-mediated resistance against RNA viruses was first reported by Zhang et al. (2018) in transgenic Arabidopsis and tobacco plants. In the study, gRNA/FnCas9 cassettes were designed to target and attack various regions in the RNA genome of *Tobacco mosaic virus (TMV)* and *Cucumber mosaic virus*. Transgenic plants with gRNA/FnCas9 constructs were found to have significantly less viral accumulation (40 to 80%) relative to the control plants. Recently discovered RNA-targeting endonuclease, Cas13, has also been used to develop plant resistance against RNA virus infection (Aman et al., 2018). For instance, tobacco plants overexpressing gRNA/Cas13a successfully targeted and inhibited the replication of *Turnip mosaic virus* (Zhang et al., 2019). Similar approaches were efficiently used for the generation of resistance in potato against *Potato virus Y* (Zhan et al., 2019) and in rice for resistance to *Southern black-streak* and *stripe mosaic viruses* (Zhang et al., 2019).

Transgenic expression of the CRISPR constructs in transgenic plants and targeting the host susceptible factors is another strategy that was also used for viral resistance. Some translation initiation factors (eIF4E, eIF(iso)4E, and eIF4G) or their isoforms are required for replication and infection of RNA viruses. Therefore, the inactivation of these susceptibility factors in plants could be used to induce resistance to a virus without damage to the plant due to their functional redundancy between the different isoforms (Cao et al., 2021). CRISPR/Cas9 has been utilized to introduce mutations into these translation initiation factors in rice and tomato (Shimatani et al., 2017). Using the same editing technique, Bastet et al. (2019) introduced a single substitution mutation into eIF4E in the plant host genome. Both studies demonstrate that mutations of these susceptibility factors are sufficient to generate viral resistance in hosts against the potyviruses *Clover yellow vein virus*.

### 3.2 Genome editing for bacterial resistance

Genome editing in plants to develop resistance against bacterial diseases is still limited in application (**Table 2**). One of the main approaches taken to develop genome-edited plants resistant to pathogenic bacteria is by the knockout of susceptibility (*S*) gene/s (Zaidi et al., 2018). These genes are transcription factors that bind to a sequence-specific promoter region and are known as effector- binding elements (EBEs). A classic example of an *S* gene is the *Mildew Resistance Locus O* (MLO) which was first associated with powdery mildew (PW) susceptibility in barley decades ago (Jørgensen, 1992). An *S* gene related to bacterial infection, *SWEET* (Sugar Will Eventually Be Exported Transporter) gene in rice is related to susceptibility to *Xanthomonas oryzae* pv. oryzae (Xoo) (Antony et al., 2010). Furthermore, *Citrus sinensis lateral organ boundary 1* (CsLOB1) gene first identified as susceptibility gene for citrus bacterial canker that caused by *Xantomonas citri* subsp. citri (Xcc) recently found to play a regulatory role with activity in cell wall remodeling and in cytokinin and brassinosteroid hormone pathways. In favor of this statement, RNAi-mediated silencing of the CsLOB1 gene developed resistance to canker disease in various citrus species (Zou et al., 2021). Contrary to silencing, overexpressing of Gretchen Hagen3 (GH3.1 and GH3.1L) genes involved in auxin signaling in citrus significantly reduced susceptibility to *Xantomonas citri* subsp. citri (Zou et al., 2019). *S* gene GE approaches such as TALEN and CRISPR/Cas9 technologies were later used to mutate effector-binding sites within the SWEET promoter and develop resistant to Xoo in rice and tomato (Zafar et al., 2020; Zeng et al., 2020; Luo et al., 2021). Similarly, in a recent study, *DOWNY MILDEW RESISTANCE 6* (*DMR6*) was mutated using CRISPR/Cas9 mediated GE to successfully produce mutant banana and tomato plants resistant against *Xanthomonas campestris* (Xcm) and other pathogenic microbes (Wang et al., 2019; Tripathi et al., 2021). Luo et al. (2021) reported immunity of rice plants to bacterial blight Xoo by employing the CRISPR/Cas9 GE system to knockout *OsPrx30* a CIII Prx precursor.

**Table 2:** Crop genome editing for bacterial resistance.

Crop	Target	Genetic Changes	Method	Status	Reference
*C. maxima*	*Xanthomonas citri subsp. citri (Xcc)*	EBE region of the LOB1 promoter in Pummelo	*Agrobacterium-mediated transformation of Pummelo epicotyls and obtaining T0*	Generation canker-resistant citrus varieties by mutation of the EBE	Jia and Wang (2020); Jia et al. (2017)
*C. sinensis Osbeck*	*Xanthomonas citri subsp. citri (Xcc)*	CRISPR/Cas9-targeted mutation in CsLOB1 promoter in citrus	*Agrobacterium mediated gRNA/Cas9 transfer and generation homozygous mutant citrus explants*	Promoter editing of CsLOB1 alone was sufficient to enhance citrus canker resistance in citrus.	Peng et al. (2017)
*C. sinensis Osbeck*	*Xanthomonas citri subsp. Citri (Xcc)*	Mutation and loss of function in *CsWRKY22*	*Agrobacterium mediated transformation of gRNA/Cas9 into epicotyl segments of orange*	Mutant orange plants showed decreased susceptibility to citrus canker	Wang et al. (2019)
*M. balbisiana*	*Xanthomonas campestris pv. musacearum (Xcm)*	Mutation in downy mildew resistance 6 (DMR6)	*gRNA/Cas9 was introduced into the embryogenic cell suspension through Agrobacterium-mediated transformation*	Musa dmr6 transgenic mutants of banana showed enhanced resistance to BXW, and did not show any detrimental effect on plant growth	Tripathi et al. (2021)
*M. domestica*	*Erwinia amylovora*	Mutation in apple *DIPM-1, DIPM-2* and *DIPM-4*	*Delivery of CRISPR/Cas9 ribonucleoproteins to the protoplast of apple cultivar*	Resistance to fire blast disease in non-transgenic but mutant apple lines	Malnoy et al. (2016)
*O. sativa*	*Xanthomonas oryzaepv. Oryzae (Xoo)*	Knockdown of the *Os8N3* in rice	*Stable transmission of CRISPR/Cas9-mediated Os8N3 gene editing without the transferred DNA)*	Transmission of mutations to generations, and enhanced resistance to Xoo in homozygous mutants.	Kim et al. (2019)
*O. sativa*	*Xanthomonas oryzae pv. oryzae (Xoo)*	Mutations in EBE of three promoters of *SWEET11, SWEET13* and *SWEET14*	*Promoter mutations were simultaneously introduced into the rice with Agrobacterium mediated transfer of gRNA/Cas9*	Stable transgenic rice lines indicated robust, broad-spectrum resistance to Xoo.	Oliva et al. (2019); Xu et al. (2019)
*O. sativa*	*Xanthomonas oryzae pv. oryzae (Xoo)*	Mutation in EBEs of *OsSWEET14* gene	*Biolistic technology was used to deliver gRNA/Cas9 into embryogenic calli of the rice*	Enhanced resistance locally isolated virulent Xoo strains	Zafar et al. (2020)
*O. sativa*	*Xanthomonas oryzaepv. Oryzae (Xoo)*	Mutation and loss of function in *OsSWEET14*	*Agrobacterium mediated stable expression of Cas9 protein together with gRNA*	Mutant rice confers strong resistance to African Xoo and Asian Xoo starins	Zeng et al. (2020)
*S. lycopersicum*	*P. syringae, P. capsici and Xanthomonas spp*	Loss of function mutation in *SlDMR6-1* gene	*Agrobacterium mediated transformation of gRNA/Cas9 tomato*	Mutants do not have detrimental effects on growth and had multiple disease resistance *P. syringae, P. capsic*i and *Xanthomonas* spp.	Paula de Toledo Thomazella et al. (2016)

### 3.3 Genome editing for fungal resistance

Loss of *S* gene function can provide more durable fungal resistance in other crop plants (**Table 3**). Functional knockouts of *StDND1*, *StCHL1*, and *StDMR6-1* susceptibility genes using CRISPR/Cas9 system generated potatoes with increased resistance against late blight (Kieu et al., 2021). Simultaneous modification of three homologues of *TaERD1* gene utilizing CRISPR/Cas9 procedure enhanced powdery mildew resistance in wheat, caused by the biotrophic pathogen *Blumeria graminis* f. sp. *tritici* (Bgt) (Zhang et al., 2017). TALENs system was used to modify Mildew resistant LOCUS (MLO) encoding proteins which repress powdery mildew defence in wheat. TALEN-induced mutation triggered heritable broad-spectrum resistance against powdery mildew disease (Wang et al., 2014). Transgene-free powdery mildew-resistant tomato variety was generated by deleting 48 bp region from *SlMLO1* locus utilizing CRISPR/Cas9 technology. The resulting plants were indistinguishable from naturally occurring mutations having the same phenotypic characteristics (Nekrasov et al., 2017). Another study was performed on tomato *Powdery Mildew Resistance 4* (*PMR4*) gene mutagenesis through CRISPR/Cas9 which resulted in mutants with reduced but not complete loss of susceptibility to powdery mildew pathogen *Oidium neolycopersici* (Santillán Martínez et al., 2020). To define the functions of SlymiR482e‐3p gene in response to tomato wilt disease, caused by the *Fusarium oxysporum* f. sp. *lycopersici* fungus, CRISPR/Cas9 was used to knock-out the gene in a disease susceptible tomato cultivar. The resulting tomato mutants exhibited significant disease reduction (more than 90%) in SlymiR482e‐3p levels and increased resistance to the necrotrophic pathogen displaying the same phenotypic traits with the control plants (Gao et al., 2021). In *Gossypium hirsutum*, simultaneous editing of two *Gh14-3-3d* gene copies through CRISPR/Cas9 technology led to enhanced transgene-free resistance to *Verticillium dahliae* in allotetraploid cotton (Zhang et al., 2018). *Agrobacterium*-mediated transient transformation was used to introduce CRISPR/Cas9 components into cacao leaves and cotyledon cells targeting *Non-Expressor of Pathogenesis-Related 3* (*TcNPR3*) gene, a suppressor of the defense response. The edited tissues exhibited enhanced immunity against *Phytophthora tropicalis* which is a widespread fungal pathogen (Fister et al., 2018). Developing *Fusarium oxysporum* (FON) resistant watermelon varieties by traditional breeding methods is hampered by the limited FON-resistant germplasm. Knockout of *Clpsk1* gene in watermelon through CRISPR/Cas9 system conferred resistance to FON, and thus established a base to develop disease-resistant germplasm in watermelon (Zhang et al., 2020).

**Table 3:** Crop genome editing for fungal resistance.

Crop	Target	Genetic Changes	Method	Status	Reference
*C. lanatus*	*Fusarium oxysporum.*	Loss-of-function in Phytosulfokine1 *(ClPSK1)* in watermelon	*Transformation of gRNA/Cas9 to watermelon through Agrobacterium tumefaciens-mediated transformation*	Loss-of-function rendered watermelon seedlings more resistant to infection by *F. oxysporum.*	Zhang et al. (2020)
*C. papaya*	*P. palmivora*	Mutation on Extracellular cystatin-like cysteine protease inhibitor (PpalEPIC8) of papaya	*PpalEPIC8 mutants were generated using CRISPR/Cas9-mediated gene editing via Agrobacterium-mediated transformation*	Reduced pathogenicity during infection	Gumtow et al. (2018)
*G. hirsutum*	*Verticillium dahliae*	Indel mutations in negative defence gene *(Gh14-3-3d)* of cotton	*Agrobacterium-mediated transformation of gRNA/Cas9 into cotton*	Higher and heritable resistance to *Verticillium dahliae* infestation in mutant cottons	Zhang et al. (2018)
*O. sativa*	*M. oryzae*	Mutation in rice ERF Transcription Factor Gene *OsERF922*	*Agrobacterium-mediated transformation of the embryogenic calli of rice*	Enhanced resistance in mutant rice to *M. oryzae* in subsequent generations	Wang et al. (2016)
*S. lycopersicum*	*Powdery Mildew Resistance 4 (SlPMR4)*	Knock-out of the tomato *SlPMR4* gene	*Transferring CRISPR/Cas9 construct containing four single-guide RNAs (sgRNAs)to target SlPMR4*	Haustorial formation and hyphal growth were diminished but not completely inhibited in the mutants	Santillán Martínez et al. (2020)
*S. lycopersicum*	*Fusarium oxysporum f.* sp. *Lycopersici*,	Mutation in *SlymiR482e-3p*, a member of the *miR482/2118* superfamily in tomato, negatively regulating the resistance	*Agrobacterium transfer of gRNA/Cas9 into susceptible tomato cultivar*	Enhanced resistance to tomato wilt disease in edited plants	Gao et al. (2021)
*S. tuberosum*	*Phytophthora infestans*	Tetra-allelic deletion of StDND1, StCHL1, and StDMR6-1 in potato	*Agrobacterium mediated transfer of multiple gRNA/Cas9 in to potato*	Editing confers increased late blight resistance in potato	Kieu et al. (2021)
*T. cacao*	*Phytophthora tropicalis*	Deletions in Non-Expressor of Pathogenesis-Related 3 *(TcNPR3)* gene, a suppressor of the defence response	*Agrobacterium was used to introduce a CRISPR/Cas9 system into leaf tissue*	The edited tissue exhibited an increased resistance to infection with the cacao pathogen *Phytophthora tropicalis*	Fister et al. (2018)
*T. aestivum*	*Powdery mildew*	Indel mutations at the wheat Mildew-resistance locus (MLO)	*Wheat protoplasts transformation with TALEN and CRISPR vectors*	TALEN and CRISPR-induced mutation at TaMLO homeologs, confers heritable broad-spectrum resistance to powdery mildew.	Wang et al., 2014
*T. aestivum*	*Blumeria graminis f.* sp. *tritici (Bgt)*	Simultaneous modification of the three homologs of wheat enhanced disease resistance1 *(TaEDR1)*	*Biolistic transformation of gRNA/Cas9 plasmids into wheat immature embryos*	Mutant wheats were resistant to powdery mildew and did not show mildew-induced cell death.	Zhang et al. (2017)
*V. vinifera*	*Erysiphe necator and Plasmopara viticola*	Editing the *DM* and *PM* susceptibility genes in different grapevine clones	*CRISPR/Cas9 technology was used to edit DM and PM susceptibility genes*	Multiple resistance against grape wine powdery mildew and downy mildew	Giacomelli et al., 2018
*V. vinifera*	*Oomycete pathogen Plasmopara viticola*	Loss-of-function mutations in *grapevine pathogenesis-related 4 (PR4)*	*Agrobacterium mediated gRNA/Cas delivery into Thompson Seedless*	The VvPR4b knockout lines had increased susceptibility and disease symptoms of downy mildew in mutant grapevine	Li et al. (2020)

As discussed previously plants have evolved complex defense mechanisms including plant hormones such as abscisic acid, salicylic acid, jasmonic acid and ethylene. Plant ethylene responsive factors (ERF) play roles in various biotic stress responses. The ethylene responsive factor *OsERF922* was edited using CRISPR/Cas9 which led to enhanced blast resistance in rice (Wang et al., 2016). In summary, gene editing technologies can offer robust and durable resistance against the most destructive fungal pathogens confronted in crop production worldwide.

### 3.4 Genome editing for resistance of crops to parasitic weeds

Plants are autotrophic organisms using light as energy for converting carbon into carbohydrates by photosynthesis. On the other hand, some other plants have evolved specialized organs (haustorium) which attach forming vascular connections with autotrophic plants in order to absorb their water and nutrients. This heterotrophic lifestyle is known as parasitic plants/weeds and has a profound negative impact on many important crops and trees affecting these ecological systems (Hu et al., 2020). The existence of parasitic plants in lower diversified agrological systems can cause yield losses and make some land uncultivable (Fernández-Aparicio et al., 2020). Weeds tend to compete with crops for water, nutrients, and light sources. However, parasitic weeds’ haustorial connections to either the xylem or phloem directly extract water and nutrients from host plants and cause permanent damage to the crops’ life cycle (Albert et al., 2020). Traditional weed management methods tend to be ineffective, expensive and labor-intensive. Parasitic weeds generally produce large numbers of small seeds that make it difficult to detect and eradicate contamination of the soil or the crop seeds before parasitism are established. The seeds of parasitic plants have long dormancy and viability in soils and germinate after receiving the host signals (Delavault, 2020).

The discovery of the terpenoid lactones in crops (e.g., strigolactones (SLs) and sesquiterpene lactones (STLs), Xie et al., 2010; Chadwick et al., 2013) is a milestone in understanding the responses of parasitic weeds and to their hosts. Host roots synthesize trace amounts of secondary metabolites which have several important physiological processes in host plants from shoot branching to arbuscular mycorrhizal symbiosis. Terpenoid lactones were then realized to be the germination stimulants for several obligate parasitic species, including broomrapes (e.g., *Orobanche* and *Phelipanche* spp.) (Raupp and Spring, 2013; Cheng et al., 2017). The seeds of these parasitic plants germinate when they receive terpenoid lactone signals from their hosts. Thus interactions between parasitic weeds and hosts have evolved in a very specific way dependent on the detection of the presence of STLs or SLs by parasitic weeds and coordinate their germination and development with the host’s lifecycle (Spring, 2021). Reducing the quantity of such stimulant exuded by host plants was always considered to be a key factor for the host resistance achieved by inhibition of parasitic weed seed germination. CRISPR, and RNAi mediated gene silencing strategies have been used to block strigolactones (SLs) synthesis in hosts (Vogel et al., 2010; Kohlen et al., 2012; Aly et al., 2014; Dubey et al., 2017; Butt et al., 2018; Bari et al., 2019; Wakabayashi et al., 2019). In this way, the germination of seeds of parasitic plants was suppressed and almost complete resistance to parasitic weeds was achieved in genome-edited host plants.

### 3.5 Recent advances in genome editing and new potential applications for plant pathogen resistance

Base editors enable single-nucleotide changes in the genomes without cutting or removing the nucleic acid backbone. CRISPR-Mediated base editing (CBE) used a single-stranded DNA-specific cytidine deaminase fused to an inactivated Cas9 (dCas9) to convert a cytosine (C)-guanine (G) base pair to thymine (T)-adenine (A) in the target region with the help of sgRNA (Li et al., 2023). CBE have lots of potential and theoretical application that can be used for disease resistance in plants. For instance, a CBE can convert C to T (G to A in the opposite strand) precisely, turning glutamine (CAA and CAG), arginine (CGA), and tryptophan (TGG) codons into stop codons (Kuscu et al., 2017). If this precise substitution (generating a nonsense mutation) occurs in the gene of interest, it will cause premature termination of translation and abort the gene’s function. It can also be used to alter splicing mechanisms in plant species. The splicing of intronic regions highly depends on conserved 5′GT and 3′AG sequences. Theoretically, if the conserved sites mutate, it will interfere with mRNA splicing, cause mRNA mis-splicing, and eventually disrupt gene function. In addition to this loss-off function application; CBE can be also used for the gain of function in plants. In this technique introducing a targeted point mutation in a gene turns a nonfunctional SNP into a functional one. The best example of CBE-based gain of function is *Acetolactate synthase* (ALS) gene which is a key enzyme in the biosynthesis of branched-chain amino acids, making it an effective target for developing herbicides. CBE was exploited to target wheat mutants ALS with a change of C-to-T conversion at the conserved Pro174 residue and they showed herbicide resistance (Zong et al., 2017). Similarly, CBE was used to create a series of missense mutations in the *OsALS* to confer herbicide tolerance in rice (Zhang et al., 2020). ALS has also been successfully edited in other species (Chen et al., 2017; Tian et al., 2018; Veillet et al., 2019; Jiang et al., 2020). These base editor systems have been also effectively used to enhance plants’ resistance to pathogens. For instance, previous studies have indicated that a single amino acid substitution at position 441 of the recessive allele of the Pi-d2 gene resulted in the loss of resistance to rice blast (*M. oryzae*). CBE technology was successfully used to introduce a G-to-A substitution in a recessive allele of Pi-d2. The deduced protein contained an amino acid substitution, which recovered the resistance of rice to *Magnaporthe oryzae* (Ren et al., 2018). In another study, Wang et al. (2020) used the same system to target the effector binding element within the promoter of the OsSWEET14 gene in rice. The base-edited mutant rice exhibited high resistance to the leaf blight fungus. Using the CBE technique, Bastet et al. (2019) introduced a single substitution mutation into eIF4E in the plant host genome and mutations of this susceptibility factor were found to be sufficient for resistance to *potyviruses clover yellow vein virus*.

In recent years, enzymatically inactive mutant of Cas9 (dead or deactivated Cas9 -dCas9) was developed in which its endonuclease activity is non‐functional. The applications of CRISPR/dCas9 have expanded and diversified in recent years (Moradpour and Abdulah, 2020). Originally, dCas9 was used as a CRISPR/Cas9 re‐engineering tool that enables targeted expression of any gene or multiple genes through recruitment of transcriptional effector domains (promoters) without introducing irreversible DNA‐damaging mutations (**Figure 2**). dCas9 started to become a powerful tool for targeted inhibition of gene transcription in plants. dCas9 can easily directed with sgRNAs to the promoter regions of the genes and functions as a repressor or block for the transcriptional machinery, a phenomenon called CRISPR interference (CRISPRi). CRISPRi has been reported to be used for effective, stable RNA‐guided transcriptional suppression of a target gene in several plant species (Larson et al., 2013; Qi et al., 2013). sgRNA-guided CRISPR activation (CRISPRa or CRISPR-Act) systems have also been developed in plants for increased expression of target genes. In this system, various gene activator proteins were fused to the dCas9 and directed to the promoter region of the target genes with sgRNAs. Binding of CRISPRa to the target promoter region up‐regulated expression of the gene of interest in plants (Tiwari et al., 2012; Piatek et al., 2015; Li et al., 2017; Lowder et al., 2017a; Lowder et al., 2017b) (**Figure 2**). Both CRISPRa and CRISPRi technologies have not been utilized for the improvement of plant resistance to pathogens yet. However, they have a large potential and flexibility that can be used for R gene-mediated resistance in plants instead of S gene-dependent loss of function approaches.

Genome editing is widely applied via stable integration of gRNA/Cas9 construct with selective marker gene to plants’ genome. However, transgene integration in plant genomes raises important legislative concerns regarding genetically modified plants. In order to obtain transgene-free edited plants, it is necessary for the integrated foreign DNA to segregate out via selfing or crossing with wild-type plants (Gao et al., 2021). This is a labor intensive and time-consuming process, and thus not suitable for several plant species. Genome editing by using CRISPR ribonucleoproteins (RNPs) has become an attractive approach for many crop species with many advantages. In this system, a ribonucleoprotein (RNP) complex consisting of Cas9 protein and single guide RNA (sgRNA) directly delivered to protoplast cell culture via bombardment, polyethylene glycol-mediated transfection or electro-transfection. RNP-mediated genome editing can be achieved shortly after cell transfection because transcription or translation is not required. RNP complex is degraded in the cell and transgene free mutant plant lines could be obtained after regeneration. This system would become a powerful and widespread method for genome editing due to its advantages of DNA/transgene-free editing, minimal off-target effects, and reduced toxicity due to the rapid degradation of RNPs and the ability to titrate their dosage while maintaining high editing efficiency. Although RNP-mediated genetic engineering has been demonstrated in many plant species, its editing efficiency remains modest, and its application in many species is limited by difficulties in plant regeneration and selection. Although RNP-mediated genetic engineering has been demonstrated in many plant species (Zhang et al., 2021), its editing efficiency in terms of pathogen resistance in plants remain to be tested.

## 4 Impacts – risks, challenges and future perspectives

Genome editing is offering new tools and opportunities for the improvement of plant disease resistance. The development of efficient methods for its wider application in resistance breeding has the potential to create a significant impact on crop cultivation in the future. However, as with all things new, it will face some challenges to be overcome, and create potential risks that have to be taken into account.

### 4.1 Impacts on crop improvement – advantages and limitations

Plant breeding for the production of new resistant varieties using classical approaches has had limited success (Ahmad et al., 2019) due to the potential of pathogens, through recombination and/or mutation and the development of novel genotypes that are no longer sensitive to resistance genes. G E enables the production of desired pathogen/pest resistance in plants that could supplement traditional or molecular breeding methods and reduce breeding cycles. So far, the successful application of genome editing in pathogen/pest resilience and its introduction into crops has been limited by the lack of information on genome sequences in crop plants and the characterization of potential target genes. Fortunately, numerous species have been fully sequenced in last decades, enabling genome editing of many crops. Crop genetic studies have described details of crop immunity, and now identified larger numbers of potential targets for control of pathogens.

A range of plant defence mechanisms can be used by breeders to protect plants. The R gene-type of disease resistance has been exploited in traditional plant breeding and generally, it is preferred over immunity systems based on PTI as it is a qualitative resistance easier to select. However, it is less durable and pathogen populations easily adapt to overcome disease resistance (Collinge, 2020; Li et al., 2020; Li et al., 2020). Pathogen resistance obtained through *R*-genes is limited in use as *R*-gene conferred resistance is generally pathogen race specific and is overcome by the evolution of new races. Hence, susceptibility regulators of disease resistance, (*S*-genes), provide better targets for GE (Yin and Qiu, 2019). They have emerged as an alternative to *R*-genes, as *S*-genes conferring resistance are recessively inherited and editing in ‘S’ alleles through CRISPR/Cas9 exhibits more broad-spectrum and durable forms of resistance than resistance genes (R-genes) against pathogens (Yildirim et al., 2012; Van Schie and Takken, 2014) and provide crop resistance that has the potential to be more persistent in the field. Using GE techniques such as CRISPR-Cas9 or RNAi can remove or inactivate these genes and impair the pathogens’ ability to cause disease (Lapin and Van den Ackerveken, 2013). S-gene mutants can be produced in most crops without considering species barriers due to the functional conservation of S-genes across crop species. Further advances in molecular studies of main crops will also enable the discovery of novel S-genes, thus providing additional targets for GE. However, S-genes are also involved in other plant physiological processes so their inactivation could disrupt crop development. This factor may hamper the application of S-gene editing in crop improvement (Yin and Qiu, 2019).

Progress in understanding the specific processes involved in pathogen-host interactions is expected to pave the way to the employment of gene drives for the creation of crops that are immune to certain pathogens and pests, and no longer support pathogen growth (Hefferon and Herring, 2017). Gene drive systems applied for eradicating malaria vector mosquitoes (Kyrou et al., 2018) could be used as a model for control of sexually inheriting crop pests and pathogens. However, the application of gene drives could also lead to changes in entire pest and pathogen communities thus affecting current ecosystems which need to be considered before wider application of this technology in crop improvement (Hefferon and Herring, 2017). Finally, the recent publication and subsequent retraction of the article of Zhang et al. (2021) and Zhang et al. (2022) reporting the design of a gene drive based on CRISPR Cas9 that targets specific genetic elements in *Arabidopsis*, shows that the application of gene drives, although promising, is still not in ready for wide use in crop improvement.

### 4.2 Risks and challenges

If gene editing involves transgenics (*i.e.*, SDN-3) then there is a consensus that the products are considered as GMOs by most regulatory authorities or as Novel Plants by the Canadian authorities. SDN-1 and SDN-2 plants usually do not contain foreign DNA and so are not regulated as GMOs in many/most countries, an exception being the European Union (EU) (Rostocks, 2021). In many countries non-transgenic gene editing is considered a development of conventional breeding and so regulations are being developed on this basis (Jenkins et al., 2021). However, the European Commission is now proposing that plants that could be created using conventional breeding techniques are exempt from the EU GMO regulations (European Commission, 2023). This would include many genome edited plants from SDN-1 and SDN-2 as well as some cisgenic types. Thus there is some convergence of the regulations on GE plants.

As in all plant breeding processes, unintended and off target effects can also occur in gene editing, though it is argued that gene editing has higher levels of precision and targeting so that they will occur much less frequently than in conventional and other types of mutation breeding, as well as the transformation techniques using DNA as the transfecting agent (Okita and Delseny, 2023). In addition, there may be reduced genetic stability in GE plants in target or associated *loci*. These effects may compromise the efficacy and durability of enhanced pest and disease resistance. It is therefore important that plant breeders test for and identify any pleiotropic, off target or stability effects using both molecular techniques and phenotypic, field studies.

Some attempts to develop viral resistance using CRISPR have not been successful and presented some of the disadvantages of CRISPR. Mehta et al. (2019) was not able to induce resistance to *African cassava mosaic virus* in GM cassava plants that overexpressed gRNA/Cas9 constructs targeting the viral transcription activator and replication enhancer protein. Similar results were also recorded when the CRISPR/Cas9 system was used to block coding sequences of *TYLCV, MeMV*, and *Cotton leaf curl Kokhran virus* (Ali et al., 2016). Instead, both studies found that CRISPR editing produced new mutant variants which were probably due to repair post-cleavage. Thus, GE may present some risks, due to the production of new virus variants.

Procedures for risk assessment of GE plants have been proposed by Eckerstorfer et al. (2021) and Lema (2021) and discussed by EFSA (Naegeli et al., 2020). Obviously, changes to nutritional quality of plants should be assessed but Eckerstorfer and co-workers (Eckerstorfer et al., 2021) also stressed that novel or enhanced traits of GE plants should be considered for their environmental impacts. Factors to be considered include any non-target effects, including considering changes in pathogenicity and weediness of pathogens associated with GE crops and consequences of changes to fitness and invasiveness of GE plants and hybridizing species.

### 4.3 Conclusions and future challenges

Due to its various advantages, CRISPR/Cas technology has become the technology of choice in wide aspects of scientific research for a short time period. However, there are still bottlenecks and challenges for its wider implementation and usage. In most life-science laboratories in the world, this technology has found its place in fundamental research, prevalently in animal cells in comparison to plants. The future research directions of GE in plants should evolve in aspects of gene delivery, resolving modalities to expand high throughput editing strategies, discovering new Cas enzymes to lower the limitations in specific gene targeting and enhancing the regenerative capacity and stability of transformation effects. One of the future research directions in genome editing, that will be of crucial importance in increasing current low transformation efficacy is finding a solution of breaking the recalcitrance in diverse plant species in tissue culture. To overcome this, alternative methods like virus-induced GE (VIGE) and nanotechnology-based GE, have recently been developed to avoid the need for *de novo* regeneration from tissue culture. However, to increase the adoption of these technologies, it will be important to overcome the limitations set by the size of the Cas enzyme (Cardi et al., 2023).

GE plants with improved pest and disease resistance have the potential to introduce more durable resistance and thus contribute towards more sustainable pest and disease management. More durable resistance mechanisms can be produced by down regulating S-genes and up regulating genes that identify pathogens, inhibit infection and reduce the virulence of pathogens by inhibiting development using genetic modifications, gene editing, RNAi and gene drives. Combining these mechanisms and managing levels of exposure to pests and pathogens in IPM can make major contributions to improving the sustainability of agricultural production, particularly in response to climate change, and to achieving Unite Nations Sustainable Development Goals and National/EU policy objectives for agriculture and the environment. Strategies for exploiting GE crops have been extensively reviewed by Bartlett et al. (2023). Of particular importance is the improvement of traits such as tolerance to biotic stresses and herbicides, self-compatibility to allow for self-pollination and inbreeding, lower content of toxic compounds as steroidal glycoalkaloids, browning free fruits and tubers, and improvements in starch quality (Tuncel and Qi, 2022).

Public perceptions and attitudes to the use of GE technologies for producing crops and foods are critical for the introduction of GE produce into food production and supply chains and require clear communication of the benefits and risks. These issues have been extensively discussed by several authors e.g., Strobbe et al. (2023) and Will et al. (2023).

It is important that appropriate and science-based policies and regulations are in place that allow rapid assessment of the risks of the products from these new breeding techniques. This will create preconditions for responsible usage of GE technology and its wider application. In addition, plant breeders and crop variety evaluators should be able assess the net contribution that new varieties can make to sustainable farming systems considering present and future requirements in relation to climate change and other externalities influencing food production and supply chains.

## Author contributions

EA, JS, and DM developed manuscript outline. All authors contributed to writing and editing. KY prepared Figure and Tables. DM, EA, JS, and KY did final editing and manuscript preparation. All authors contributed to the article and approved the submitted version.
